# Esterification of Acetic Acid by Flow-Type Membrane Reactor with AEI Zeolite Membrane

**DOI:** 10.3390/membranes13010111

**Published:** 2023-01-14

**Authors:** Yuma Sekine, Motomu Sakai, Masahiko Matsukata

**Affiliations:** 1 Department of Applied Chemistry, Waseda University, 513 Wasedatsurumaki-cho, Shinjuku-ku, Tokyo 162-0041, Japan; 2 Research Organization for Nano & Life Innovation, Waseda University, 513 Wasedatsurumaki-cho, Shinjuku-ku, Tokyo 162-0041, Japan; 3 Advanced Research Institute for Science and Engineering, Waseda University, 513 Wasedatsurumaki-cho, Shinjuku-ku, Tokyo 162-0041, Japan

**Keywords:** zeolite, membrane reactor, flow reactor, plug flow, esterification, acetic acid, ethyl acetate, AEI

## Abstract

AEI-type zeolite membrane for dehydration was prepared, and a flow-type membrane reactor for the esterification of acetic acid and ethanol by AEI membrane was developed. A synthesized AEI membrane had suitable molecular sieving property for gas separation (H_2_/*i*-butane and CO_2_/CH_4_) and pervaporation (H_2_O/acetic acid). AEI membrane showed H_2_O permeance of 6.2 × 10^−7^ mol m^−2^ s^−1^ Pa^−1^ with a separation factor of 67 at 363 K for the equimolar mixture of H_2_O/acetic acid. AEI membrane maintained stable performance under acidic conditions. The yield of ethyl acetate at 363 K in a flow-type membrane reactor with AEI membrane successfully exceeded the equilibrium of 69.1%, reaching 89.0%. The flow rate of feed solution strongly affected the conversion of acetic acid and the space–time yield (STY) of ethyl acetate. Due to the more significant proportion of water selectively removed from the reaction system at a lower feed flow rate, the thermodynamic equilibrium shifted significantly, resulting in higher conversions. In contrast, STY increased with increasing feed flow rate. Our flow-type membrane reactor exhibited a relatively large STY of 430 kg m^−3^ h^−1^ compared with the batch-type membrane reactor previously reported.

## 1. Introduction

Esters produced by esterification are widely used in chemicals such as fragrances, solvents, pharmaceutical products, and polymer materials. Esterification of organic acid and alcohol is reversible; thus, product yield is constrained by thermodynamic equilibrium. Currently, esterification is carried out in batch reactors using an excess amount of alcohol to increase acid conversion. Therefore, a considerable amount of energy is consumed to purify the ester from the remaining alcohol and water obtained as a by-product [1].

Dehydration membrane reactors have drawn attention as a novel energy-saving process for esterification for recent decades. Employing dehydration membranes to remove water from the reaction system increases the conversion level achievable beyond the thermodynamic limitation without excess alcohol. Consequently, one can expect that energy consumption for the purification process after reaction is reduced as well as the improvement of the one-path yield of products.

Zeolite is well-known as a builder, catalyst, and adsorbent [2]. Zeolite membranes have been widely used as dehydration membrane from organic solvents [3,4,5]. In addition, some types of zeolite membrane showed excellent dehydration properties and stability in acidic solutions [6,7,8]. For these reasons, membrane reactors for esterification using zeolite membranes have been previously studied and demonstrated the yield improvement of ester beyond the thermodynamic equilibrium [9,10,11,12,13].

8-ring zeolites are one of the promising membrane materials for dehydration from H_2_O/alcohol because of their small pore size. In particular, LTA- and CHA-type zeolites are well known as great dehydration membranes because these zeolites have a 3-dimensional pore network and large pore volume leading to high permeation properties. Recently, AEI-type zeolite, which has a very similar structure to CHA-type zeolite, has also been expected to be a novel 8-ring zeolite. The synthesis method and crystallization behavior of AEI powdery crystals have been widely studied for the last ten years [14,15,16,17,18,19]. Several synthetic routes have been developed from zeolites [14,15,16,17,18] and amorphous [19] as raw materials. In addition, catalytic performance for NH_3_-SCR and partial oxidation of methane have been studied [20,21].

On the other hand, a few literatures about the preparation method for AEI membrane were reported. *Zhan* et al. reported a preparation method of AlPO_4_-18, an aluminophosphate with AEI structure, membrane for CO_2_/CH_4_ separation [22]. In their study, the AlPO_4_-18 membrane showed the separation factor for CO_2_/CH_4_ of 75 at 303 K by molecular sieving effect. *Hasegawa* et al. also reported the high separation performance of AEI membrane for H_2_O/ethanol mixture [23]. 

Recently, flow reaction has attracted attention in the synthesis of fine chemicals because a flow-type reactor exhibits high productivity and is easy to control the production volume. Although the batch-type membrane reactor for esterification has been previously studied, such a flow reactor was rarely reported. For example, Iglesia et al. successfully demonstrated the effectiveness of a flow membrane reactor for esterification using MOR zeolite membranes [12]. However, the effect of operation conditions of membrane reactor on the level of conversion and space time yield is still an open question. In this study, we fabricated a tubular AEI-type zeolite membrane for dehydration and developed a flow-type membrane reactor incorporating this AEI membrane for the esterification of acetic acid and ethanol. Using this reactor, the operating conditions of the flow-type membrane reactor for esterification were explored, particularly the effect of flow rate on the ethyl acetate yield. The results confirmed the efficacy of the flow-type membrane reactor for dehydration.

## 2. Experimental

### 2.1. AEI Powdery Crystal Preparation

AEI powder crystal was synthesized following the previous report by Nakazawa et al. [18]. FAU-type zeolite powder with Si/Al of 5.55 (HSZ-350HUA, TOSOH, Japan), colloidal silica (LUDOX AS-40, Sigma-Aldrich, Germany), sodium hydroxide (>97%, Kanto chemical, Japan), and 1,1,3,5-tetramethylpiperidinium hydroxide (TMPOH, 20 wt% aqueous solution, SACHEM, US) were used as an aluminum source, a silicon source, mineralizer, and organic structure-directing agent (OSDA).

A synthesis solution having the composition of 1.0SiO_2_: 0.017Al_2_O_3_: 0.15Na_2_O: 0.17TMPOH: 6.0H_2_O was prepared as follows. The mixture of 8.87 g of TMPOH, 8.96 g of 8.78 wt% NaOH aqueous solution, and 8.12 g of colloidal silica was heated up to 353 K while stirring. The mixture evaporated until the total weight of the mixture became less than 13.6 g. After that, 1.0 g of FAU-type zeolite powder dried at 373 K was added to the resultant mixture. Distilled water was also added until the total weight of the mixture became 16.1 g. The mixture was homogeneously stirred by hand using a PTFE rod. The homogeneous mixture was poured into a Teflon-lined stainless-steel autoclave and hydrothermally treated at 433 K for 72 h. After hydrothermal treatment, white sedimentation was procured by filtration. Finally, we obtained AEI powdery crystal.

A slurry was prepared using AEI crystal for seeding in membrane preparation as follows. The obtained AEI crystal was ground using a uniaxial ball-mill at 200 rpm for 24 h. After grinding, 20 g of distilled water was added to a ground crystal to form a suspension. Centrifugation took place for the suspension at 3500 rpm for 15 min, and then the supernatant was recovered as a seed slurry. The solid content in a slurry was adjusted at 5.0 g L^−1^ by adding a given amount of distilled water. All operations for slurry preparation were performed at room temperature, ca. 293 K.

### 2.2. Membrane Preparation

A membrane was synthesized by a seed-assisted method. Porous α-Al_2_O_3_ tube was used as a support (O.D., 10 mm; I.D., 7 mm; average pore size of surface layer, 150 nm; Noritake, Japan). Seeded support was prepared by a dip-coating method using seed slurry. Both ends of the tubular support were plugged with PTFE caps. The support was dipped in a seed slurry for 1 min and then withdrawn vertically at approximately 3 cm s^−1^. After drying at 343 K for 2 h, the dip-coating process was repeated. Calcination at 773 K for 3 h was performed for seeded support to suppress the seed crystals’ peeling from the support surface.

The synthesis solution with a molar composition of 1.0SiO_2_:0.010Al_2_O_3_:0.1Na_2_O: 0.50TMPOH:29.2H_2_O. A synthesis solution was prepared by mixing sodium aluminate (31.0~35.0% Na_2_O, 34.0~39.0% Al_2_O_3_, Kanto Chemical, Japan), tetraethyl orthosilicate (TEOS, Sigma-Aldrich, Germany), sodium hydroxide (>97%, Kanto Chemical, Japan), and TMPOH. The composition of synthesis solution was determined by reference to a previous report [19].

A synthesis solution was prepared as follows. The mixture of 11.0 g of distilled water, 0.149 g of sodium aluminate, 0.364 g of NaOH, and 21.3 g of TMPOH was stirred for 20 min. A total of 11.1 g of TEOS was slowly added to the mixture. The mixture was aged at 293 K for 24 h prior to crystallization. The seeded support was placed vertically in a Teflon-lined stainless-steel autoclave, filled with the aged synthesis solution, and then hydrothermally treated at 433 K for 24 h. After crystallization, the autoclave was quenched by flowing tap water. The membrane was washed with boiling water for 8 h. After washing, the membrane dried overnight at 383 K.

The obtained membrane was calcined to remove OSDA at 573 K for 60 h under an O_2_ stream containing O_3_ of 200 ppm. The calcined membrane was used for separation measurements.

### 2.3. Characterization for Powder and Membrane

Prepared powdery crystals and membrane were characterized by using XRD (Ultima-IV, Rigaku, Japan), FE-SEM (S-4800, Hitachi, Japan) with EDS (X-max, Oxford instruments, UK). FE-SEM observation was carried out with the accelerating voltage of 1 kV and a current of 5 μA. For elemental analysis by EDS, the accelerating voltage and current were adjusted to 5 kV and 20 μA, respectively.

### 2.4. Gas Separation and Pervaporation Tests

The permeation property of membrane was evaluated in pervaporation and gas separation. A tubular membrane was placed inside a stainless-steel module and sealed using graphite cylindrical rings at both ends. The effective membrane area was 6.28 cm^2^. The compositions of permeate liquid and gas were determined using GC equipping a TCD-detector (GC-2014, Shimadzu, Japan). The separation factor, *α*, of each separation test was determined as follows:

*α* [-] = (*Xi Xj*^−1^) (*Zi Zj*^−1^)^−1^ (1)

where *Xi* and *Xj* are molar fractions of components *i* and *j* [-] in permeate, respectively. *Zi* and *Zj* are molar fractions in feed.

Pervaporation was carried out using the equipment schematically shown in Figure S1a. The feed mixture was pumped to the outer surface of tubular membrane. Permeate was vacuumed and captured by a cold trap with liquid nitrogen. Flux, *J*, was calculated by the following equation:

*Ji* [mol m^−2^ s^−1^] = *Wi Mi*^−1^ *A*^−1^ (3600)^−1^ (2)

where *Wi* and *Mi* are captured weight per an hour [g h^−1^] and molecular weight [g mol^−1^] of component *i*, respectively. *A* corresponds to a membrane area [m^2^].

Figure S1b schematically shows the equipment for gas separation. The feed gas mixture was fed to the outer surface, and permeated the gas inside of support was swept by flowing gas, Ar. The permeation side was kept at atmospheric pressure in gas separations. Permeance, *Π*, was determined as follows:

(3) *Πi* [mol m^−2^ s^−1^ Pa^−1^] = *Ji ΔP*i^−1^

where *ΔPi* is the partial pressure difference of component *i* between the feed and permeate sides [Pa].

### 2.5. Membrane Reactor Test for Esterification in the Flow-Type Reactor

Membrane reactor tests were conducted using the same equipment as the pervaporation tests. Amberlyst-15 was introduced as a catalyst between the membrane surface and the inside wall of the module. The feed solution flowed in the catalyst bed at a constant flow rate. A membrane reactor test was carried out at 363 K. An equilibrium composition mixture of acetic acid, ethanol, ethyl acetate, and H_2_O at 363 K (0.15EtOH:0.15AcOH:0.35AcOEt:0.35 H_2_O) was used as the feed solution. The solutions that flowed out from both permeate and retentate sides were analyzed for their compositions.

The yield of ethyl acetate, *Y*, and material balance, *B*, were calculated by molar flow rates of each component, *Fi*, according to the following equations:

(4) $$Y\;[\%]=\frac{F_{AcOEt,\;Permeate+Retentate\;}\left[\mathrm{mol}\;h^{-1}\right]}{F_{AcOEt,Feed\;}\left[\mathrm{mol}\;h^{-1}\right]+F_{AcOH,Feed\;}\left[\mathrm{mol}\;h^{-1}\right]}\times 100$$

(5) $$B\;[\%]=\frac{F_{AcOEt,\;Permeate+Retentate\;}\left[\mathrm{mol}\;h^{-1}\right]+F_{AcOH,\;Retentate+Permeate\;}\left[\mathrm{mol}\;h^{-1}\right]}{F_{AcOEt,Feed\;}\left[\mathrm{mol}\;h^{-1}\right]+F_{AcOH,Feed\;}\left[\mathrm{mol}\;h^{-1}\right]}\times 100$$

## 3. Results and Discussion

### 3.1. Membrane Characterization

**Figure 1:**
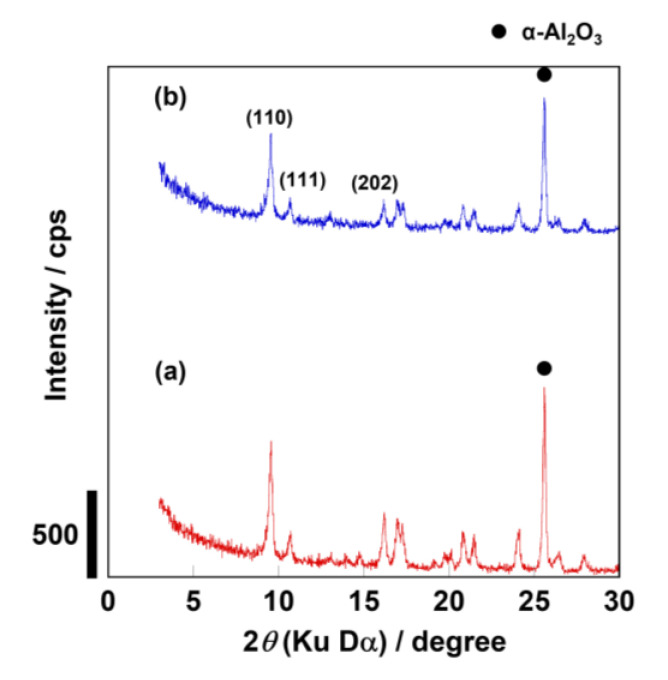
XRD patterns of membrane synthesized (**a**) before and (**b**) after calcination.

Figure 1 shows the XRD patterns of membranes before and after calcination. There were no apparent peaks other than those corresponding to the AEI-type zeolite and α-alumina in the as-made sample before a calcination step. In addition, a relative crystallinity was calculated from the area of the reflection peak appearing at 2*θ* = 9.56 degrees. Based on the crystallinity of the sample before calcination as 100%, the crystallinity of the membrane after calcination was 88%. Although the crystallinity slightly decreased, the crystal structure of AEI-membrane was maintained after the calcination.

**Figure 2:**
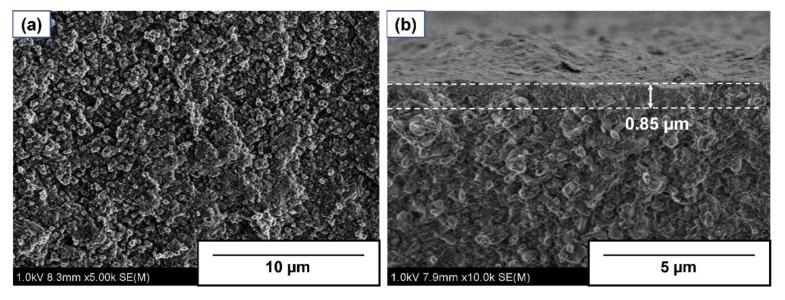
Typical FE-SEM images of the (**a**) surface and (**b**) cross-sectional views of AEI membrane.

Figure 2 shows the typical FE-SEM images of the surface and cross-sectional views of AEI membrane. A continuous polycrystalline membrane was observed from the surface view. The polycrystalline membrane was composed of small crystals with several hundred nm. The Si/Al ratio of the membrane surface was evaluated at 2.9 with EDS analysis. This value was substantially lower than that of AEI powdery crystal of ca. Sixteen synthesized under kindred conditions [23]. Therefore, the Si/Al ratio of the crystal layer would be underestimated by EDS because of the signal from α-Al_2_O_3_ support beneath a very thin membrane.

The thickness of a continuous layer on the support surface was determined at ca. 1 μm from the cross-section view. In addition, membrane thickness was also evaluated by using EDS analysis. Si was detected from a depth of 5 μm or more from the outer surface, indicating that zeolite crystals were also formed inside the porous support.

### 3.2. Gas Separation Property of AEI Membrane

The molecular sieving property of AEI membrane was evaluated by gas separation tests for equimolar H_2_/i-butane and CO_2_/CH_4_ mixtures. Table 1 lists the results of gas separation tests. In the H_2_/*i*-butane separation test, AEI membrane exhibited the H_2_ permeance of 4.44 × 10^−7^ mol m^−2^ s^−1^ Pa^−1^ and the separation factor of 202 at 373 K. In addition, AEI membrane was thermally stable up to 573 K, e.g., a separation factor was 248 at 573 K with the H_2_ permeance of 5.63 × 10^−7^ mol m^−2^ s^−1^ Pa^−1^. For CO_2_/CH_4_ separation, CO_2_ selectively permeated through AEI membrane with a permeance of 6.39 × 10^−7^ mol m^−2^ s^−1^ Pa^−1^ and a separation factor of 74 at 298 K.

The results of the gas separation tests listed in Table 1 suggested that the synthesized AEI membrane had suitable molecular sieving property. The kinetic diameters of molecules used in separation tests, H_2_, CO_2_, CH_4,_ and *i*-butane are 0.29, 0.32, 0.38, and 0.48 nm, respectively. H_2_ and CO_2_ readily enter and diffuse in the micropore of AEI because the size of micropore is 0.38 nm. Although CH_4_ is able to enter and diffuse in this micropore as well, the diffusion rate of CH_4_ would be one magnitude smaller than those of H_2_ and CO_2_. In contrast, *i*-butane cannot enter the AEI micropore because of its bulky molecular size. For these reasons, AEI membrane showed selectivity of H_2_ and CO_2_ from the mixtures of H_2_/*i*-butane and CO_2_/CH_4_.

### 3.3. Separation Property of AEI Membrane for H_2_O/AcOH Mixture

A separation test for the binary mixture of H_2_O/acetic acid was carried out to evaluate the potential of AEI membrane for the esterification membrane reactor. The separation test was conducted at 363 K for an equimolar mixture of H_2_O and acetic acid in pervaporation mode.

**Figure 3:**
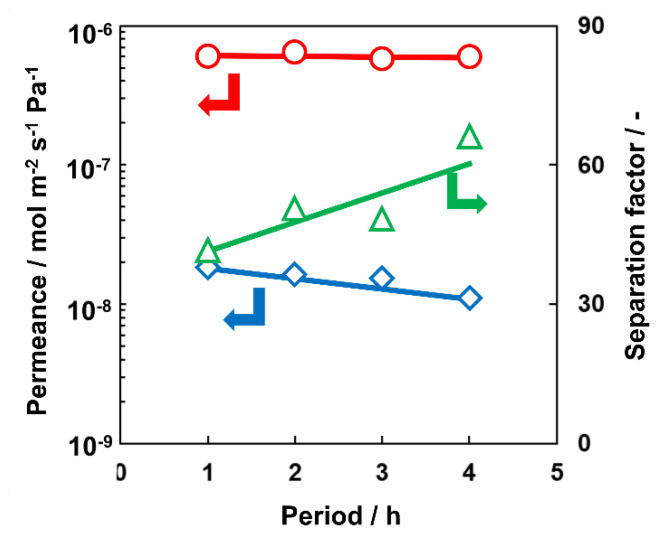
Permeation and separation performance of AEI membrane for the equimolar mixture of H_2_O/acetic acid at 363 K in pervaporation mode. ○, H_2_O; ◊, acetic acid; ▵, separation factor.

Figure 3 shows the time course of H_2_O and acetic acid permeances over the pervaporation test. H_2_O permeance was almost constant at 6.2 × 10^−7^ mol m^−2^ s^−1^ Pa^−1^ over 4 h. In contrast, the permeance of acetic acid slightly decreased during the separation test from 2.0 to 1.2 × 10^−8^ mol m^−2^ s^−1^ Pa^−1^. As a result, the separation factor of H_2_O against acetic acid increased from 40 to 67. 

Because acetic acid was not able to enter the micropore of AEI crystal due to the bulky molecular size, acetic acid would penetrate through the small amount of inter-crystalline defects such as pinholes and cracks in AEI membrane.

H_2_O and acetic acid could permeate through small inter-crystalline defects by surface diffusion. In surface diffusion, adsorption amounts of H_2_O and acetic acid strongly affect each permeation flux. The flux of acetic acid changed with time course, possibly because competitive adsorption took time to reach equilibrium. In contrast, H_2_O flux was not affected by surface diffusion in the inter-crystalline pathway, since most of the H_2_O penetrated through micropores of AEI zeolite.

Finally, AEI membrane had stable permselectivity for H_2_O/AcOH mixture, suggesting that the membrane exhibited a good potential for an esterification membrane reactor.

### 3.4. Esterification Membrane Reactor for AcOH with EtOH

AEI membrane was applied for a flow-type membrane reactor for the esterification of acetic acid and ethanol. The esterification in the membrane reactor was carried out at 363 K. The mixture of acetic acid, ethanol, ethyl acetate, and H_2_O was used as the feed solution. The composition of the mixture was adjusted as equilibrium at 363 K, e.g., acetic acid: ethanol: ethyl acetate: H_2_O = 0.155: 0.155: 0.345: 0.345. The flow rate of feed solution was changed from 0.05 to 0.15 mL min^−1^, resulting in the contact time from 816 to 272 s.

Here, we defined the dehydration ratio as follows:

Dehydration ratio [%] = *F*_H_2_O,permeate_/(*F*_H_2_O,permeate_ + *F*_H_2_O,retentate_) × 100 (6)

Equilibrium should be shifted by the selective removal of H_2_O in the esterification membrane reactor. Hence, how much amount of H_2_O was removed by the membrane was very important to understand the effect of membrane reactor. The dehydration ratio corresponds to the ratio of H_2_O removed from the reaction system to H_2_O generated in the reactor. In other words, the dehydration ratio was determined by the rates of catalytic reaction and dehydration by membrane.

**Figure 4:**
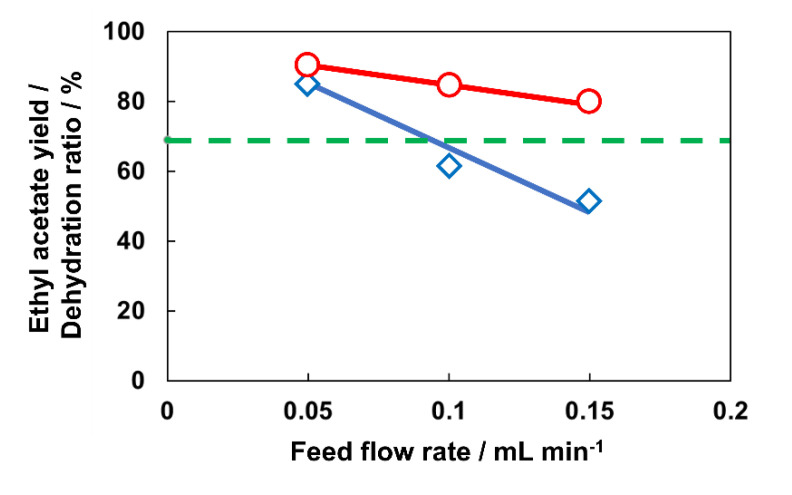
The relationship between the yield of ethyl acetate and dehydration ratio with different feed flow rates. ○, Ethyl acetate yield; ◊, dehydration ratio. The dashed line showed the equilibrium yield.

**Figure 5:**
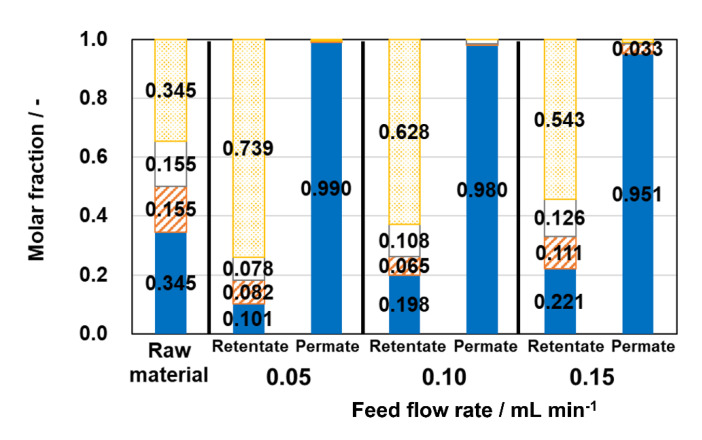
Molar compositions of permeates and retentates in membrane reactor tests with different feed flow rates. Solid bar, H_2_O; shaded bar, ethanol; open bar, acetic acid; dotted bar, ethyl acetate.

Figure 4 shows the yield of ethyl acetate and dehydration ratio with different feed flow rates. Higher ethyl acetate yield and dehydration ratio were exhibited at a lower feed flow rate. For example, the yield of ethyl acetate and the dehydration ratio reached 89.0 and 84.6%, respectively. This yield overwhelmed the 69.1% yield of ethyl acetate at equilibrium under the conditions used. It is noted that material balances were in the range of 97.4–100% in all cases. Figure 5 shows the compositions of permeates and retentates in the membrane reactor tests with different feed flow rates. In all cases, the ratio of H_2_O in retentate decreased, and that in permeate increased compared with the composition of feed material. At the same time, the ratio of acetic acid and ethanol in the retentate decreased by further reaction, and that of ethyl acetate increased.

The compositions of permeates in the membrane reactor were almost occupied by H_2_O, suggesting that AEI membrane selectively removed H_2_O from the quaternary mixture. Therefore, equilibrium in the reactor was shifted by the selective removal of H_2_O, resulting in high ethyl acetate conversion.

The dehydration ratio increased with decreasing feed flow rate because of longer contact time in the flow-type reactor. Due to the more significant proportion of water selectively removed from the reaction system at a lower feed flow rate, as shown in Figure 4, the thermodynamic equilibrium shifted significantly, resulting in higher conversions. In addition, the increase in the dehydration ratio with increasing contact time also suggested that the reaction rate is enough larger than the dehydration rate. If the reaction rate is lower than the dehydration rate, the dehydration ratio would reach the ceiling of ca. 100% and did not be influenced by contact time.

**Figure 6:**
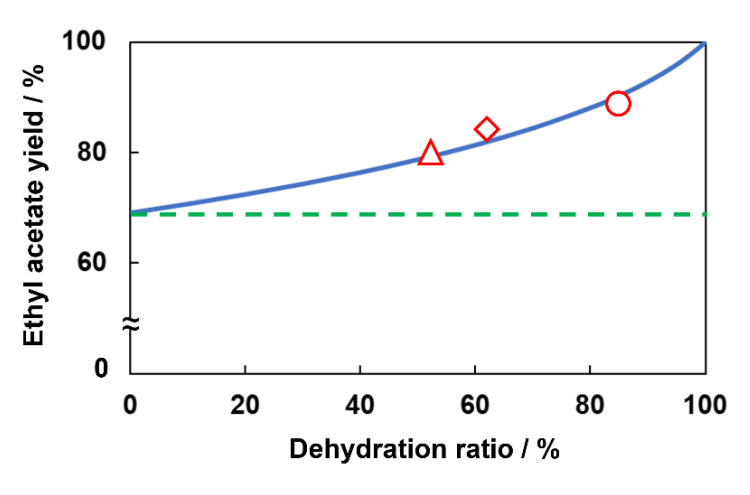
The relationship between dehydration ratio and ethyl acetate yield.

Figure 6 shows the dehydration ratio and ethyl acetate yield. The blue line shows the calculated yield at equilibrium with the selective removal of H_2_O. Here, we assumed that only water penetrated through the membrane to calculate the equilibrium yield. In addition, the green dashed line exhibited equilibrium yield without dehydration in a conventional reactor. The experimental results shown as red plots had a good agreement of calculated equilibrium conversion, indicating that the reaction rate and separation performance were good enough for this membrane reactor. To improve the yield of ethyl acetate by membrane reactor, the dehydration ratio should be improved: e.g., improvement of H_2_O permeation performance and/or increasing contact time are essential.

The result of the membrane reactor test showed the potential of flow-type membrane reactor for the esterification of acetic acid and ethanol was exhibited clearly. In addition, the balance between the rates of reaction and dehydration in a membrane reactor can be evaluated by the dehydration ratio. When the dehydration ratio is low, the H_2_O permeation performance of a membrane should be improved. On the other hand, if dehydration reaches the ceiling of 100%, the reaction rate should be improved by the development of catalyst.

### 3.5. Space-time Yield in Membrane Reactor

We compared the space time yield of ethyl acetate in our membrane reactor with those in some previous reports. The following equation calculated space–time yield:

STY [kg m^−3^ h^−1^] = *W_ethyl acetate_ V*^−1^ (7)

where *V* corresponds to reactor volume [m^3^]. To estimate the effectiveness of membrane reactor, we evaluated only the amount of ethyl acetate generated over equilibrium in this equation.

Table 2 lists the STY of ethyl acetate in membrane reactors. It should be noted that the STY of previous reports was calculated by us using reactor volume, ethyl acetate yield or acetic acid conversion, and flow rate in the flow reactor or feed weight in the batch reactor.

From Table 2, flow-type membrane reactors exhibited higher STY and lower conversion compared with batch-type membrane reactors [11,24,25]. Contact time can be prolonged in the batch-type reactor, resulting in increased conversion. In contrast, a flow-type reactor has high producibility based on contentious operation, as described in the introduction. In other words, STY strongly depends on the flow rate. In the case of the previous flow-type membrane reactor by MOR-type zeolite membrane, the feed flow rate was an order of magnitude smaller than that of our experiment, possibly because the H_2_O flux through their MOR membrane was limited [12]. Our flow-type membrane reactor exhibited a large STY of 430 kg m^−3^ h^−1^ even in the lower acetic acid conversion of 79 % because of the relatively large flow rate achieved by the high H_2_O flux through the AEI membrane.

**Table 2:** Space time yields of ethyl acetate in membrane reactors.

Reactor Type	Membrane	Flow Rate /mL min^−1^	Equilibrium Yield/%	Ester Yield/%	STY /kg m^−3^ h^−1^	Ref.
Batch (343 K)	Na-A	-	60.0	90	190	[11]
Flow (358 K)	MOR	0.005	72.4	82	38.7	[12]
Batch (363 K)	MOR	-	77.0	97	15.4	[24]
Flow (403 K)	CHA	0.0076	80.0	90	8.29	[25]
Flow (363 K)	AEI	0.05	69.1	89	237	This work
		0.10		84	403	
		0.15		79	430	

## 4. Conclusions

We developed an AEI-type zeolite membrane and a flow-type membrane reactor for the esterification of acetic acid and ethanol by AEI membrane.

Prepared AEI membrane had suitable molecular sieving property. For example, the membrane exhibited CO_2_ permeance of 6.39 × 10^−7^ mol m^−2^ s^−1^ Pa^−1^ with a separation factor of 74 for the equimolar mixture of CO_2_/CH_4_. AEI membrane showed stable dehydration performance for H_2_O/acetic acid mixture in pervaporation mode: e.g., H_2_O permeance through AEI membrane was 6.2 × 10^−7^ mol m^−2^ s^−1^ Pa^−1^ with a separation factor of 67.

The ethyl acetate yield was successfully increased using a flow-type membrane reactor. Using a flow-type membrane reactor with AEI membranes, the yield reached 89.0% at 363 K, exceeding the equilibrium yield of 69.1%. In addition, the effect of feed flow rate on ethyl acetate yield was studied. Higher ethyl acetate yields were obtained because more water was removed at lower flow rates: ethyl acetate yields with the feed flow rate of 0.05 and 0.15 mL min^−1^ were 89 and 79 %, respectively. However, STY increased with increasing feed flow rate. Our flow-type membrane reactor exhibited a large STY of 430 kg m^−3^ h^−1^ even with the lower acetic acid conversion of 79% due to the relatively large flow rate. These results demonstrated the effectiveness of a flow-type membrane reactor with AEI membrane.

## Figures and Tables

**Table 1:** Gas separation test results for AEI membrane.

System	Temperature/K	H_2_ or CO_2_ Permeance/10^−7^ mol m^−2^ s^−1^ Pa^−1^	Separation Factor/-
H_2_/*i*-butane (50/50 mol%)	373	4.44	202
H_2_/*i*-butane (50/50 mol%)	573	5.63	248
CO_2_/CH_4_ (50/50 mol%)	298	6.39	74
CO_2_/CH_4_ (50/50 mol%)	373	3.41	35

## Supplementary Materials

The following supporting information can be downloaded at: https://www.mdpi.com/article/10.3390/membranes13010111/s1, Figure S1: Apparatus for membrane reactor test; Figure S2: Apparatus for gas separation test; Figure S3: EDS-mapping of AEI membrane.
