# Supplementary material for: Esterification of Acetic Acid by Flow-Type Membrane Reactor with AEI Zeolite Membrane
Source: Membranes (Basel). 2023 Jan 14;13(1):111. doi: 10.3390/membranes13010111 (PMC9864284; doi:10.3390/membranes13010111)
Supplement: Supplementary file 1 [file membranes-13-00111-s001.zip › membranes-2099604-supplementary.pdf]

Supplementary Materials

Figure S1 shows an apparatus for membrane reactor test. Pervaporation test was also carried out by the same equipment without catalyst.

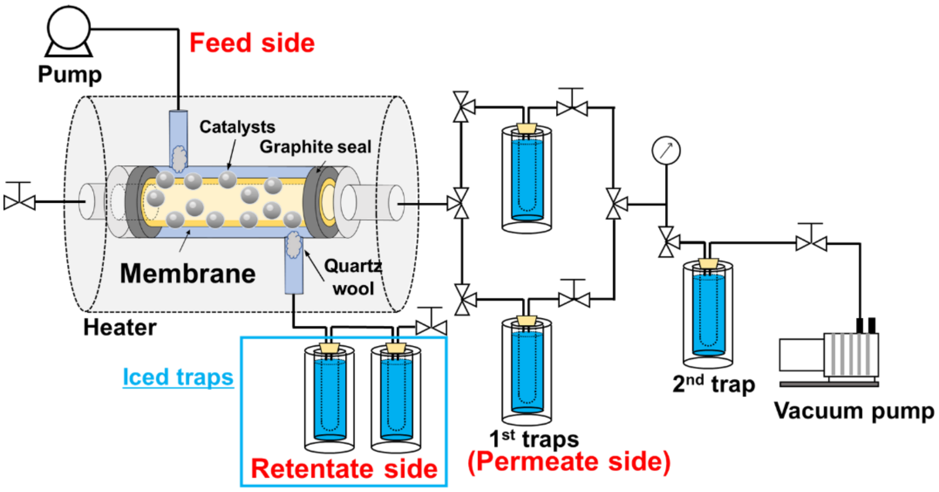

Figure S1. Apparatus for membrane reactor test.

Figure S2 shows an apparatus for gas separation test.

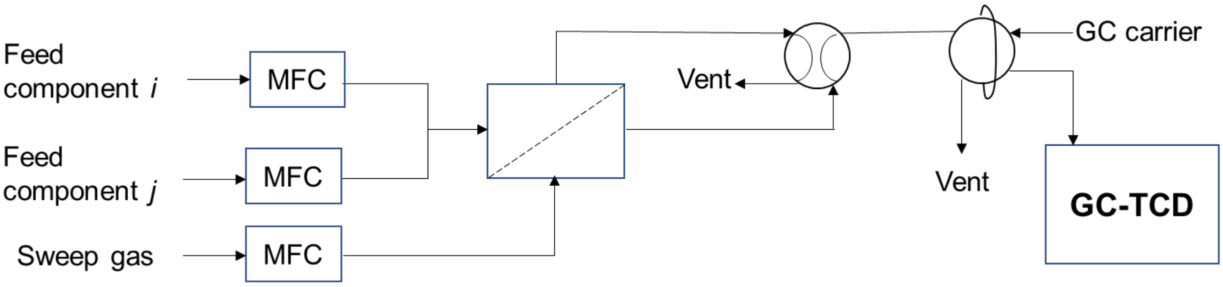

Figure S2. Apparatus for gas separation test.

Figure S3 shows the EDX-mapping of AEI membrane. The Si/Al ratio of the crystal layer would be underestimated by EDS because of the signal from α-Al_2O_3 support beneath a very thin membrane. In addition, Si was detected from the inside, indicating that zeolite crystals were formed not only outer surface but also inside the porous support.

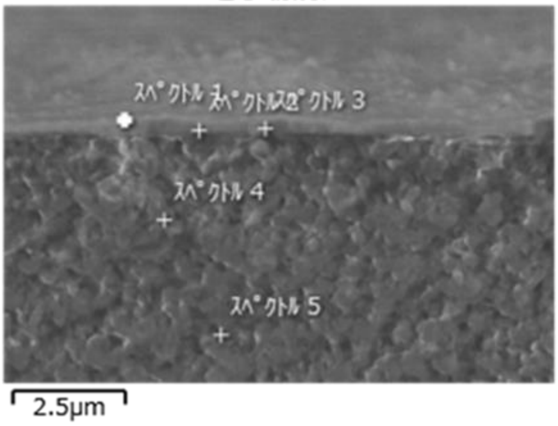

Spectrum number	Si/Al ratio
1	2.89
2	0.415
3	2.03
4	1.45
5	0.838

Figure S3. EDS-mapping of AEI membrane.
